# Lack of sustained improvements in erectile function following low-intensity extracorporeal shockwave therapy correlate with decreases in corporal brain-derived neurotropic factor: a pilot study and prospective clinical trial

**DOI:** 10.1093/sexmed/qfaf107

**Published:** 2026-01-22

**Authors:** Skye Coffey, Vy Nguyen, Ashley N Matthew, Bridget S Kastelberg, Maria E Teves, Mina Ghatas, Adam P Klausner, Ryan P Smith, Sarah C Krzastek

**Affiliations:** School of Medicine, Virginia Commonwealth University, Richmond, VA 23298, United States; School of Medicine, Virginia Commonwealth University, Richmond, VA 23298, United States; Department of Urology, Virginia Commonwealth University, Richmond, VA 23298, United States; School of Medicine, Virginia Commonwealth University, Richmond, VA 23298, United States; Department of Obstetrics and Gynecology, Virginia Commonwealth University, Richmond, VA 23298, United States; Department of Urology, Virginia Commonwealth University, Richmond, VA 23298, United States; Department of Urology, Virginia Commonwealth University, Richmond, VA 23298, United States; Division of Urology, Central Virginia VA Health Care Systems, Richmond, VA 23249, United States; Department of Urology, University of Virginia, Charlottesville, VA 22309, United States; Department of Urology, Virginia Commonwealth University, Richmond, VA 23298, United States; Division of Urology, Central Virginia VA Health Care Systems, Richmond, VA 23249, United States

**Keywords:** extracorporeal shockwave therapy (ESWT), erectile dysfunction (ED), angiogenesis, neurogenesis

## Abstract

**Background:**

Low-intensity extracorporeal shockwave therapy (Li-ESWT) is thought to treat erectile dysfunction (ED) by stimulating neovascularization and nerve regeneration as demonstrated in animal models by histologically increased angiogenesis and neuronal-related growth factors, though corresponding human studies are limited.

**Aim:**

We hypothesized that Li-ESWT results in appreciable increases in growth factors in human tissues, and in this proof-of-concept study we aimed to determine whether markers for neovascularization and nerve regeneration can be detected in the corporal blood of men following Li-ESWT treatment.

**Methods:**

Patients were prospectively enrolled in a clinical trial of Li-ESWT for ED. Patients received 12 bi-weekly Li-ESWT treatments of 0.2 mJ/mm^2^ at 5 Hz, 1500 shocks delivered per treatment, with follow up at 1-2 weeks, 4-6 weeks, 3 months, and 6 months post-treatment. Cavernosal penile blood samples were obtained prior to treatment and at each visit post-treatment. The concentrations of endothelial nitric oxide synthase (eNOS), neuronal nitric oxide synthase (nNOS), vascular endothelial growth factor (VEGF), and brain-derived neurotropic factor (BDNF) in penile plasma samples were measured using enzyme-linked immunosorbent assay with specific commercial kits, following the protocols provided by the manufacturer.

**Outcomes:**

eNOS, nNOS, VEGF, and BDNF were detectable and demonstrated changes in cavernosal plasma samples following Li-ESWT treatment.

**Results:**

Twenty-five patients completed all five study visits. Mean patient age was 63. Mean baseline International Index of Erectile Function-Erectile Function score prior to treatment was 14.24 (±1.21). Corporal plasma samples were analyzed for eNOS, nNOS, VEGF, and BDNF using the enzyme-linked immunosorbent assay. Levels of eNOS, nNOS, and VEGF showed an upward trend following treatment but did not reach significance. BDNF levels were noted to decrease.

**Clinical Implication:**

Corporal blood aspirates may function as surrogates for histological studies to understand effects of Li-ESWT at the tissue level in humans.

**Strengths and Limitations:**

To our knowledge, this is first the molecular study in human tissues to attempt to quantify neurogenesis and neovascularization in penile tissue following Li-ESWT for ED. Although our sample size is small, we believe this represents a promising first step in understanding the effect of Li-ESWT at a tissue level in men.

**Conclusion:**

The clinical significance of our findings is currently unknown, but markers of neovascularization and neurogenesis are detectable in corporal plasma and may change following Li-ESWT.

ClinicalTrials.gov  **ID** NCT04720755

## Introduction

Erectile dysfunction (ED) is a chronic inability to achieve or sustain a penile erection satisfactory for sexual performance. It is one of the most common medical conditions of adult male patients and can significantly impact the quality of life of the patient and their partner(s). Many treatment options exist, but most treatments are aimed at managing the symptoms of ED and do not treat the underlying cause which for many men is attributed to blood vessel and nerve dysfunction over time.^1^

Low-intensity extracorporeal shockwave therapy (Li-ESWT) is a promising treatment for ED. Extensive research has been conducted on Li-ESWT, examining its effectiveness in various conditions such as ischemic myocardial dysfunction, ischemic tissue necrosis, skin burns, muscle disorders, bone defects, nerve lesions, and Peyronie's disease, in addition to ED.^2-11^ While the exact mechanism is still not fully understood, Li-ESWT utilizes low-intensity shockwaves to induce shear stress on targeted tissues, initiating a cascade of biochemical responses. These responses include the release of specific growth factors, stimulation of cell proliferation, tissue regeneration, neovascularization, angiogenesis, and improved blood supply.^12^

Recent studies focusing on erectile tissue have shown promising results in diabetic rats, where Li-ESWT promoted the regeneration of neuronal nitric oxide synthase (nNOS)-positive nerves, endothelium, and smooth muscle within the penis.^13^^,^^14^ These improvements may be attributed to the recruitment of endogenous stem cells within the penile tissue. For instance, Lin et al. demonstrated that Li-ESWT increased the presence of endothelial cells within subtunical spaces in the penile erectile tissue.^15^ Additionally, other research suggests that Li-ESWT can regulate important cellular signaling pathways, including protein kinase RNA-like endoplasmic reticulum kinase/activated transcription factor, Wnt/β-catenin, and extracellular-signal-regulated kinase. Activation of these pathways promotes the differentiation of stem cells into endogenous penile erectile cells.^12^^,^^16^

Since the 2010 study by Vardi et al., various other studies have reported significant clinical improvement in vasculogenic ED following Li-ESWT treatment. However, many of these studies relied on patient-reported outcomes as a means to evaluate improvements in erectile function subsequent to treatment.^17^ Although subjective patient-reported questionnaires are validated for accuracy, the utilization of self-reported outcomes is susceptible to bias and placebo effect and fails to portray objective changes at the tissue level, thus potentially inadequately evaluating the overall treatment impact. There has therefore been a push to demonstrate more objective improvements in erectile function following Li-ESWT. In 2020, Huang et al. reported improvements in nocturnal penile tumescence and rigidity in 35 patients treated with 2500 shocks of 0.09 mJ/mm^2^ at 5 Hz once per week for 4 weeks.^18^ No improvements were observed in right or left cavernosal artery peak systolic velocity (PSV), end diastolic velocity (EDV), or resistive index (RI) following treatment. This is in contrast to other studies which have shown improvements in cavernosal artery hemodynamics. In 2017, Kalyvianakis and Hatzichristou treated 16 patients with sham treatment and 30 patients with 1500 shocks of 0.09 mJ/mm^2^ twice per week for 3 weeks, followed by 3 weeks without treatment, and another session of twice per week for 3 weeks. Patients in the treatment group experienced an increase in mean PSV by 4.5 cm/s as compared to 0.6 cm/sin the sham group (*P* < .001).^19^ Similarly, Goldstein et al. recently reported a reduced EDV and improvements in measurements of hypoechoic areas by grayscale ultrasound of penile erectile tissue in 22 patients treated with Li-ESWT, suggesting overall objective improvements in erectile function with treatment.^20^

Molecular studies regarding the mechanism of action of Li-ESWT at the tissue level are even more sparse. Prior studies in animal models have demonstrated increased expression of markers of neovascularization and angiogenesis including endothelial nitric oxide synthase (eNOS), neuronal nitric oxide synthase (nNOS), vascular endothelial growth factor (VEGF), and brain-derived neurotropic factor (BDNF).^21-24^ Studies in humans remain limited, largely due to the inability to perform histological studies on living human penile tissue. However, these markers are detectable in serum in circulation.^25-27^

As expression of eNOS, nNOS, VEGF, and BDNF have been shown to change following Li-ESWT in animal models, we hypothesized that these markers would be expressed within corporal blood in the rigid state and may act as a surrogate for histological studies to assess neovascularization and neurogenesis within the penile tissues. We conducted a proof-of-concept pilot study to determine if eNOS, nNOS, VEGF, and BDNF are measurable in corporal blood aspirates, and if changes in levels of these markers are detectable following Li-ESWT treatment. We hypothesize that if changes in these markers are detectable, this method of study may act as a surrogate for penile biopsy and histologic study in live patients.

## Methods

### Study design

Full approval was granted by the local institutional review board, and the study was registered on ClinicalTrials.gov. Participants were screened for eligibility and were prospectively enrolled. Men between the ages of 40-80 years old, with known or suspected vasculogenic ED for ≥6 months, in an active sexual relationship with ≥2 sexual attempts per month presenting for evaluation of ED in our outpatient, urology clinic were screened for inclusion. Additional inclusion criteria included International Index of Erectile Function-Erectile Function (IIEF-EF) scores of 8-25, Erection Hardness Scores (EHS) ≥1, total testosterone of 300-1000 ng/dL, and hemoglobin A1c ≤8% at the time of enrollment. Men with ED due to neurogenic or psychogenic causes, prostatectomy, pelvic surgery, or pelvic radiation within the preceding 12 months, anatomic malformations including Peyronie’s disease, international normalized ratio > 2.5, or taking blood thinners other than aspirin, were excluded. All participants signed a written informed consent prior to initiation of any study visits. Patients taking phosphodiesterase-5 inhibitors were required to stop this medication for 1 month prior to all study visits.

Changes in Doppler parameters were a secondary outcome of a primary study powered to detect a clinically meaningful change in IIEF-EF questionnaire score of 4 points. Power analysis was conducted by a biostatistician using the software nQuery Advisor 4.0. A paired *t*-test of the mean difference in the IIEF-EF score from baseline to post-treatment was used as the endpoint. Assuming a significance level of .05 and a two-tailed test, a 4-point improvement with a standard deviation of 5 would need a sample size of 19 to show statistical significance with a power of 90%. We proposed a sample size of 24 to protect the validity of the study should there be dropouts over the 6-month study time. Twenty-five patients were ultimately enrolled and completed all study visits.

### Li-ESWT treatment

**Figure 1:**
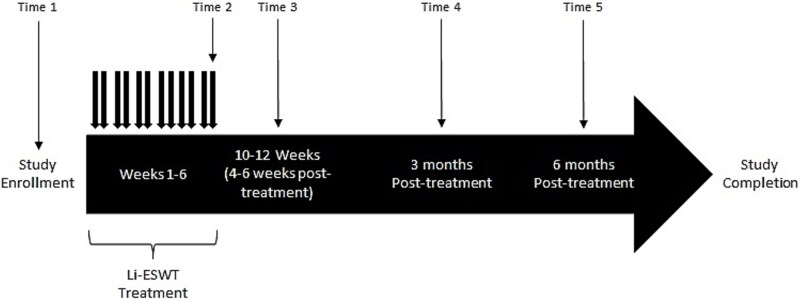
Study design.

Patients were treated with focused linear shockwaves using the Storz Duolith SD1 T-Top >> ultra << machine. Patients received twice-weekly treatments of 0.2 mJ/mm^2^ over the right and left penile shaft (500 shocks per site), and bilateral crura (250 shocks per site), for a total of 3000 shocks per week, for 6 weeks (total of 18 000 shocks) (Figure 1). Treatment protocol was selected based on protocols implemented at similar academic institutions in attempt to reduce variability across treatment protocols. All treatments were administered by a single fellowship-trained urologist with prior training and experience using the equipment.

### Study time point procedures

Participants completed validated questionnaires including the IIEF-EF domain, Sexual Encounter Profile questions 2 and 3, Global Assessment Questions, and EHS. After completion of questionnaires, penile Doppler was performed as previously described,^28^ measuring PSV, EDV, and RI in the flaccid state. Intracavernosal injection of alprostadil was administered to induce a rigid erection. Strength of alprostadil medication varied based on baseline erectile function and ranged from 2.5 to 20 mcg. Each participant’s dose of alprostadil remained unchanged for each study visit. Penile Doppler was repeated 10 min following injection. A cavernosal blood sample was then obtained via a separate lateral proximal shaft corporal stick using a 23Gx3/4″ × 12″ BD Vacutainer *UltraTouch* Push Button Blood Collection Set and 3 mL BD Vacutainer PST Gel and Lithium Heparin^N^ (LH) blood collection tube. Blood samples were analyzed by enzyme-linked immunosorbent assay (ELISA) as described below. These questionnaires and procedures were performed prior to Li-ESWT treatments, immediately following completion of treatments, 4-6 weeks following treatments, 3 months following treatments, and 6 months following treatments, for a total of five study visits (Figure 1). To minimize bias, intracavernosal injections, penile Dopplers, and all shockwave treatments were administered by the same fellowship-trained Urologist for all participants at all study visits.

### ELISA analysis of plasma samples

Quantification of biomarkers in human plasma was performed using the ELISA method. Plasma samples isolated from cavernosal blood were added to microplates pre-coated with specific antibodies for the target protein and incubated for a specified duration at room temperature or 37°C. Unbound substances were then removed through washing steps. A biotin-conjugated secondary antibody or similar detection reagent was added, followed by further incubation. After additional washes, a substrate solution (eg, 3,3',5,5'-Tetramethylbenzidine (TMB)) was applied to enable colorimetric detection. Finally, a stop solution was added, and absorbance at 450 nm was measured using a microplate reader. The colorimetric signal was proportional to the concentration of the target protein, which was quantified using a standard curve generated from known concentrations. Details specific to the quantification of each biomarker are outlined below.

#### Endothelial nitric oxide synthase

eNOS quantification in human plasma was performed using the Human eNOS/NOS3 ELISA kit (Invitrogen, catalog # EH169RB). Plasma samples (100 μL) were added to microplates pre-coated with anti-eNOS antibodies and incubated for 2.5 h at room temperature. After washing, a biotin-conjugated secondary antibody was added and incubated for 1 h, followed by a streptavidin-HRP solution for 45 min. Colorimetric development was achieved using TMB substrate, and absorbance at 450 nm was measured. eNOS concentrations were determined via a standard curve (0.4-100 ng/mL).

#### Neuronal nitric oxide synthase

nNOS quantification was performed using the Human nNOS ELISA kit (Novus Biologicals, catalog # NBP2-80252). Plasma samples (100 μL) were incubated in wells pre-coated with anti-nNOS antibodies for 90 min at 37°C. After adding a biotin-conjugated secondary antibody and washing, an avidin-HRP solution was applied. Substrate reagent was incubated, and absorbance at 450 nm was measured. nNOS concentrations were calculated using a standard curve (0.16-10 ng/mL).

#### Vascular endothelial growth factor

VEGF quantification was conducted using the Human VEGF ELISA kit (Abcam, catalog # Ab222510). Plasma samples (50 μL) were incubated in wells with anti-VEGF antibodies for 1 h at room temperature. Following washes, TMB substrate was added, and absorbance at 450 nm was measured. VEGF concentrations were determined using a standard curve (12.5-4000 pg/mL).

#### Brain-derived neurotrophic factor

BDNF quantification was performed using the Total BDNF Immunoassay kit (R&D Systems, catalog # DBNT00). Plasma samples (50 μL) were incubated with assay diluent and BDNF-specific antibodies for 2 h at room temperature. After washing, a conjugate solution was applied, followed by substrate incubation. Absorbance at 450 nm was measured, and BDNF concentrations were calculated using a standard curve (15.6-1000 pg/mL).

### Data analysis

A test for normality was performed using criteria of Skewness <2 and Kurtosis <7, which demonstrated a non-normal distribution. Therefore, to normalize the data, plasma concentrations of eNOS, nNOS, VEGF, and BDNF at each study time point were analyzed as fold change from baseline (ie, from Study Time Point #1). Results were reported as mean ± standard error. Bonferroni correction was utilized to reduce the risk of Type 1 error, and a *P*-value <.0125 was reported as significant. Paired two-tailed *t*-test was used for statistical analysis to compare results at each time point to baseline. Additional paired two-tailed t-test analysis was conducted after grouping patients by baseline IIEF-EF severity (score 22-25 mild, 17-21 mild–moderate, 11-16 moderate, and 6-10 severe). Repeated-measures Analysis of Variance (ANOVA) was used to calculate differences in fold change for each time point between patients based on age group (age 49-60, *N* = 12; age 61-70, *N* = 8; age 71 = 80, *N* = 5), and for each time point between patients based on baseline IIEF-EF severity. Regression analysis was conducted to assess for correlation between BNDF fold change and IIEF-EF score.

## Results

Twenty-nine patients were prospectively enrolled in this study. Two patients elected to withdraw prior to initiation of study visits, one patient was withdrawn following initial study visit upon diagnosis of Peyronie’s disease, and one patient was withdrawn following the second study visit due to a non-study related medical event. Twenty-five patients completed all study visits. No adverse events were reported by any study participant at any follow up time point. Patient demographics are listed in Table 1**.**

**Table 1:** Patient demographics.

	***N***	**Mean (±SE)**	**Range**
	25		
Age		61 ± 1.62 years	49-78 years
Race			
Black	11		
White	13		
Hispanic	1		
Total serum testosterone		522 ± 32.45 ng/dL	
Baseline IIEF-EF score		14.24 ± 1.21	
Mild (IIEF-EF ≥22)	3		
Mild–moderate (IIEF-EF 17-21)	6		
Moderate (IIEF-EF 11-16)	10		
Severe (IIEF-EF 8-10)	6		
Time from end of treatment to study time point			
EOT to Study Time #2		11.7 ± 1.13 days	
EOT to Study Time #3		6.7 ± 0.25 weeks	
EOT to Study Time #4		3.4 ± 0.08 months	
EOT to Study Time #5		6.3 ± 0.08 months	

*Abbreviation*: SE: standard error; IIEF-EF: International Index of Erectile Function-Erectile Function Domain; EOT: end of treatment.

**Figure 2:**
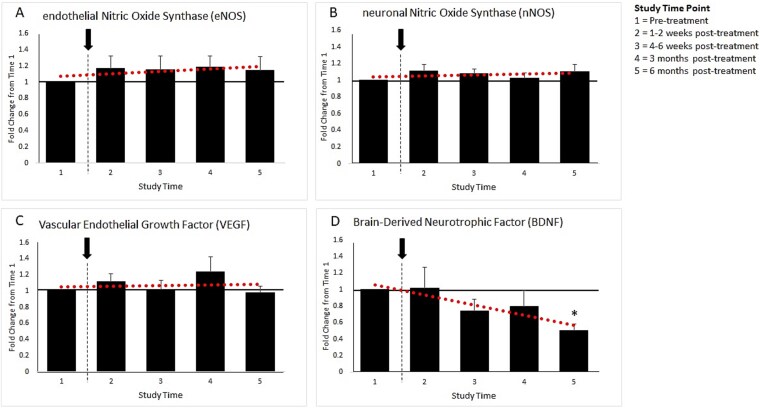
Penile corporal plasma levels of (a) endothelial nitric oxide synthase (eNOS), (b) neuronal nitric oxide synthase (nNOS), (c) vascular endothelial growth factor (VEGF), and (d) brain-derived neurotrophic factor (BDNF), at each study time point. Corporal blood aspirates were obtained prior to Li-ESWT, then following Li-ESWT at 1-2 weeks, at 4-6 weeks, at 3 months, and at 6 months. Arrow indicates Li-ESWT treatments twice per week for 6 weeks. Values reported as mean fold change from study time #1 ± standard error. ^*^P < .001.

*eNOS:* Overall, eNOS levels increased by 1.17 ± 0.15 fold at Study Time #2, 1.15 ± 0.17 fold at Study Time #3, 1.18 ± 0.14 fold at Study Time #4, and 1.15 ± 0.16 fold at Study Time #5, though increases did not achieve statistical significance (Figure 2A). When analyzed by baseline ED severity or age group, there were no statistically significant fold changes at any time point within each group or between groups.

*nNOS:* Overall, nNOS levels increased by 1.11 ± 0.08 fold at Study Time #2, 1.08 ± 0.06 fold at Study Time #3, 1.03 ± 0.06 fold at Study Time #4, and 1.11 ± 0.08 fold at Study Time #5, though increases did not reach statistical significance (Figure 2B). When analyzed by baseline ED severity, there were no significant fold changes at any time point within each group or between groups. Similarly, no significant difference was noted at any time point within groups or between groups by age.

*VEGF:* Overall, VEGF levels increased by 1.11 ± 0.10 fold at Study Time #2, by 1.02 ± 0.11 fold at Study Time #3, by 1.24 ± 0.19 fold at Study Time #4, and decreased by 0.98 ± 0.08 fold at Study Time #5 (Figure 2C). When analyzed by baseline ED severity, there were no statistically significant fold changes at any time point within each group or between groups. However, several trends were noted. Patients with severe ED (IIEF-EF 8-10) experienced fold increases ranging from 1.21 ± 0.21 to 1.42 ± 0.29 at each study time point. No significant differences were noted between ED severity groups or age groups at each time point. Fold change increased greatest in patients aged 71-80 (1.63 ± 0.39).

**Figure 3:**
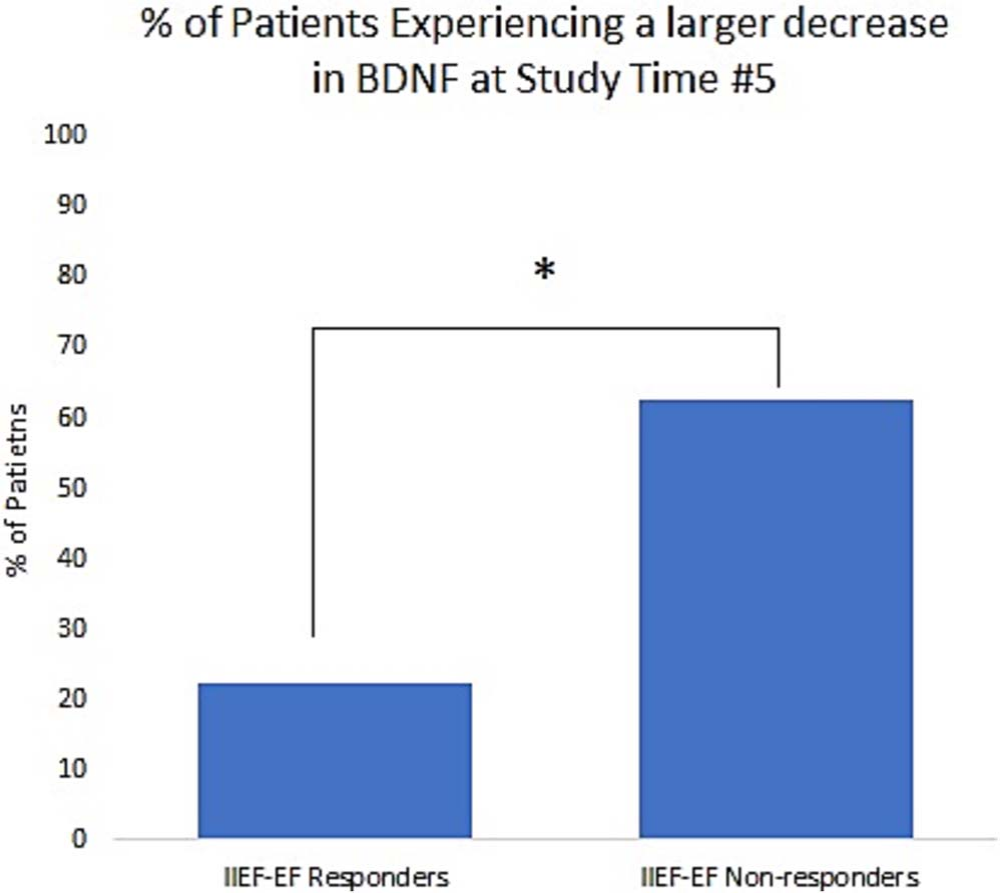
Percentage of patients with the largest BDNF fold decreases, using a threshold of median BDNF fold change of 0.42 at Study Time #5. Patients were characterized as treatment responders if they averaged a ≥4-point increase in IIEF-EF scores across all post-treatment study time points. Median BDNF fold change was 0.42. ^*^P = .03.

*BDNF:* Overall, BDNF levels increased by 1.02 ± 0.25 fold at Study Time #2, decreased by 0.74 ± 0.14 fold at Study Time #3, decreased by 0.80 ± 0.19 fold at Study Time #4, and decreased by 0.51 ± 0.07 fold at Study Time #5 (*P* < .001) (Figure 2D). When analyzed by baseline ED severity, patients with mild ED (IIEF-EF score ≥ 22, *N* = 3) did not experience statistically significant changes at any of the study time points. Patients with mild–moderate ED (IIEF-EF score 17-21, *N* = 6) initially experienced a slight fold-increase in BDNF at Study Times #2 and #3, followed by a decrease of 0.52 ± 0.18 at Time #4 and 0.42 ± 0.17 at Time #5 which approached but did not reach significance. Patients with moderate ED (IIEF-EF score 11-16, *N* = 10) experienced decreases across all time points, with a significant fold-decrease of 0.68 ± 0.08 (*P* < .01) at Study Time #3. Interestingly, BDNF was significantly decreased for patients with severe ED (IIEF-EF 8-10) at all study time points (*P* < .01). When comparing fold changes at each time point by baseline ED severity or by age no significant differences between groups were noted. Given the large fold-decrease in BDNF levels, particularly at Study Time #5 and among patients with more severe baseline ED, additional analysis was conducted to determine if fold change in BDNF correlated with subjective changes in IIEF-EF scores across the study. Regression analysis was conducted to assess the relationship between BDNF fold change and IIEF-EF score. *R*^2^ was <0.1, suggesting no clear relationship. Mean IIEF-EF scores across all post-treatment study time points were then calculated for each patient. Patients were characterized as Li-ESWT treatment responders if change in mean IIEF-EF score was ≥4 (*N* = 9). Median BDNF fold change at Study Time #5 was calculated to be 0.42. Of the 9 treatment responders, 7 (77.8%) experienced a fold decrease of ≤ the median. However, of the 16 treatment non-responders, only 6 (37.5%) experienced a fold-decrease of ≤ the mean (*P* = .03, Figure 3).

## Discussion

This study provides the first evidence of quantifiable changes in molecular markers, including eNOS, nNOS, VEGF, and BDNF, at the tissue level in human subjects. In our study, eNOS, nNOS, and VEGF levels showed trends of increase across study time points, consistent with findings in animal studies. Interestingly, BDNF levels decreased significantly following Li-ESWT, particularly in patients with more severe baseline ED, counter to what has been demonstrated previously. At face value, this finding seems counterintuitive but may reflect a more complex underlying signaling pathway.

Li-ESWT has emerged as a promising treatment for men with ED, with numerous studies reporting subjective improvements in erectile function among human subjects.^3^^,^^8^ Despite these clinical advancements, the molecular mechanisms underlying Li-ESWT remain poorly understood. Current evidence suggests that its therapeutic effects involve changes in factors associated with angiogenesis and neurogenesis.^29-31^ Previous animal studies have demonstrated improvements in eNOS, nNOS, VEGF, and BDNF following shockwave therapy. For instance, Sokolakis et al. showed increased eNOS and VEGF concentrations in rat models of ED post-shockwave therapy.^22^ Similarly, other animal studies corroborate these findings, indicating significant improvements in molecular markers associated with angiogenesis and neurogenesis.^21^ Our study was originally powered to detect a clinically significant change in IIEF-EF score of 4 points, and is therefore likely underpowered to detect statistically significant changes in biomarkers. Here, we demonstrated trends of increase in eNOS, nNOS, and VEGF levels across study time points, though fold changes did not reach statistical significance, again, likely due to underpowering. It remains to be determined what constitutes a clinically significant fold change. As above, our study was powered to detect an increase in IIEF-EF score of 4 points, generally considered clinically significant in the Men’s Health literature. Out of a 30-point questionnaire, this corresponds with an increase of 13.3%. If a 13.3% increase in serum markers is also considered clinically significant, we did find that our cohort of patients experienced a fold-increase in eNOS levels of >13% across all study time points. However, results should be interpreted with caution in the setting of underpowering and small sample size, particularly within subset analyses.

A key component of erectile function is neural stimulation, which facilitates smooth muscle relaxation and subsequent penile tumescence.^1^ BDNF, a molecular marker of neurogenesis, is known to regulate neuronal development, maturation, and survival following injury. Consequently, BDNF has been explored as a potential therapeutic target for neurodegenerative diseases.^32^ Wang et al. demonstrated a significant increase in BDNF levels in rat models with nerve injury following Li-ESWT.^23^ In our study, BDNF levels began to decrease by Study Time #3 and showed a significant decrease at Study Time #5. This counterintuitive finding may reflect complex cell signaling pathways. A prior study of the relationship between nitric oxide synthase and BDNF suggest that nitric oxide may increase cyclic GMP and ultimately downregulate BDNF secretion.^33^ It is also possible that the decrease in BDNF noted in this study is due to a longer study time period. The study by Wang et al. measured BDNF levels out to 26 days, versus the 6 months in our study. Perhaps the initial increase in BDNF noted in prior studies fails to capture an ultimate downregulation of the BDNF secretion pathway with longer-term NOS stimulation.

Reduced BDNF levels may play a role in inflammation, aging, neurodegenerative disorders, and psychiatric conditions such as bipolar disorder and major depressive disorder, suggesting that systemic or localized neurophysiological factors might influence these results.^34-36^ Given the potential effect of aging on decreasing levels of BDNF, we performed additional analysis on BDNF levels in our study to determine if there was a relationship between BDNF changes based on age. While BDNF levels decreased significantly across study time points particularly for patients with more severe baseline disease and for the entire cohort by Study Time #5, when comparing fold changes at each time point by baseline ED severity or by age no significant differences between groups were noted. The clinical implications of a decrease in this context remain unclear and warrant further investigation. Importantly, our study demonstrates that these biomarkers are detectable in penile corporal serum using ELISA, representing a step forward in understanding molecular changes following Li-ESWT at the tissue level. The methodology proposed in this study may allow investigators to identify biomarkers associated with Li-ESWT treatment response and represents an important first step in understanding the mechanism of action of Li-ESWT in humans.

As Li-ESWT appears to be most effective in patients with mild-to-moderate ED,^37^ we also wished determine if fold changes differed by baseline ED severity. When analyzed by pre-treatment IIEF-EF score of ≥22 (mild), 17-21 (mild–moderate), 11-16 (moderate), or 8-10 (severe), several trends were noted. The largest fold increases in eNOS levels were seen in patients with mild or moderate disease. The largest fold increases in nNOS and VEGF levels were seen in patients with severe disease. Fold changes in BDNF initially increased slightly in patients with mild and mild–moderate ED but decreased by Time #4 for patients with mild–moderate disease and decreased in both groups by Time #5. BDNF decreased at all-time points for patients with moderate and severe ED, particularly for patients with severe ED. As above, the clinical significance of this is unknown and interpretation of these results is limited by small sample size.

While these findings offer valuable insights, certain limitations must be acknowledged. Significantly, the study's small sample size and the absence of a sham control group restrict the generalizability of the results. However, patients served as their own internal controls by comparing pre- and post-treatment measurements. We also acknowledge that exclusion criteria for enrollment did not assess for all known risk factors for ED. As such, our patient population may not reflect pure vasculogenic ED. In addition, this study was primarily a proof-of-concept pilot study to determine whether our proposed methods could detect changes in levels of molecules generally considered to be markers of neurogenesis and neovascularization. Despite potential intrapersonal variability, the consistency of the Li-ESWT protocol and molecular assays, all conducted by the same providers, minimized methodological variability.

The ability to objectively measure the effects of therapeutic interventions is invaluable. Although Li-ESWT has shown subjective improvements in ED symptoms, objective data on its mechanisms remain limited. Our study suggests that molecular markers involved in angiogenesis and neurogenesis are detectable in corporal aspirates, and Li-ESWT modulates these markers at the tissue level, providing an objective framework for future investigations. By elucidating these mechanisms, healthcare providers may gain a deeper understanding of which patients are most likely to benefit from this therapy and optimize its clinical application for the management of ED. Further studies with larger sample sizes and rigorous control groups are warranted to build on these findings and enhance our understanding of this novel therapeutic intervention.
